# Carrageenan in the Diet: Friend or Foe for Inflammatory Bowel Disease?

**DOI:** 10.3390/nu16111780

**Published:** 2024-06-06

**Authors:** Nina Kimilu, Katarzyna Gładyś-Cieszyńska, Magdalena Pieszko, Dorota Mańkowska-Wierzbicka, Marcin Folwarski

**Affiliations:** 1 Students’ Scientific Circle of Clinical Nutrition, Medical University of Gdansk, 80-211 Gdansk, Poland; 2 Department of Clinical Nutrition and Dietetics, Medical University of Gdansk, 80-210 Gdansk, Polandmagdapieszko@gumed.edu.pl (M.P.); 3 Department of Gastroenterology, Dietetics and Internal Diseases, Poznan University of Medical Sciences, 60-355 Poznan, Poland; 4 Home Enteral and Parenteral Nutrition Unit, Nicolaus Copernicus Hospital, 80-803 Gdansk, Poland

**Keywords:** inflammatory bowel disease, carrageenan, inflammation, health, food additives, microbiome

## Abstract

While the exact pathogenesis of IBD remains unclear, genetic, environmental and nutritional factors as well as the composition of the gut microbiome play crucial roles. Food additives, which are increasingly consumed in the Western diet, are being investigated for their potential effects on IBD. These additives can affect gut health by altering the composition of the microbiota, immune responses, and intestinal permeability, contributing to autoimmune diseases and inflammation. Despite the growing number of studies on food additives and IBD, the specific effects of carrageenan have not yet been sufficiently researched. This review addresses this gap by critically analyzing recent studies on the effects of carrageenan on the gut microbiota, intestinal permeability, and inflammatory processes. We searched the MEDLINE and SCOPUS databases using the following terms: carrageenan, carrageenan and inflammatory bowel disease, carrageenan and cancer, food additives and microbiome, food additives and intestinal permeability, and food additives and autoimmune diseases. In animal studies, degraded carrageenan has been shown to trigger intestinal ulceration and inflammation, highlighting its potential risk for exacerbating IBD. It can affect the gut microbiota, reduce bacterial diversity, and increase intestinal permeability, contributing to “leaky gut” syndrome. Some studies suggest that carrageenan may inhibit the growth of cancer cells by influencing the progression of the cell cycle, but the anti-cancer effect is still unclear. Carrageenan may also increase glucose intolerance and insulin resistance. Further research is needed to determine whether carrageenan should be excluded from the diet of individuals with IBD.

## 1. Introduction

The term inflammatory bowel disease (IBD) refers to diseases of the gastrointestinal tract of unknown etiology [1,2], characterized by chronic inflammation [3,4]. They progress with alternating periods of relapse and remission [5]. In IBDs, Crohn’s disease (CD) and ulcerative colitis (UC) are the most common [1]. In Crohn’s disease, the lesions are segmental and transmural and can be localized throughout the gastrointestinal tract, from the mouth to the anus, but most commonly form in the terminal ileum [6], and much less frequently in the upper gastrointestinal tract [3], while ulcerative colitis affects the mucosa of the large intestine, particularly the colon and rectum [3,7]. These diseases can occur at any age. Differences in the age of onset are observed depending on geographical region [4] and gender [8]. The most common symptoms are diarrhea [3], rectal bleeding, bowel movement urgency, and tenesmus [1]. Other common symptoms are abdominal pain, nausea, vomiting [1], decreased appetite, and weight loss [3,9]. Inflammatory markers, such as an increased erythrocyte sedimentation rate (ESR), C-reactive protein (CRP), leucocytosis, and calprotectin, indicate active inflammation [9]. Extraintestinal manifestations occur in 25–40% of IBD cases, with joint manifestations such as arthritis and ankylosing spondylitis being the most common. Skin changes such as erythema nodosum or pyoderma gangrenosum, liver complications including primary sclerosing cholangitis, and ocular complications are less frequent [1,9]. In 2019, there were approximately 4.9 million IBD cases worldwide, with the highest number in China, followed by the USA [10] and 1.3 million cases in Europe in 2020 [8]. The pooled global prevalence of CD and UC per 100,000 person-years in 2017–2023 was 186.18 and 255.92, respectively [11]. 

**Figure 1:**
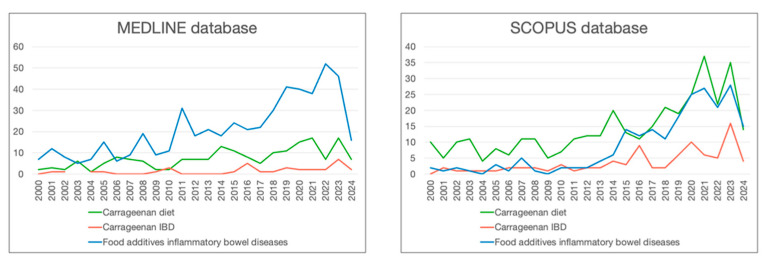
Studies on carrageenan and food additives in MEDLINE and SCOPUS databases from 2000 to 2014.

The etiology of IBD is multifactorial. It is not yet fully understood, but key factors include genetic factors [3,4,12], dietary factors [13,14,15], an inappropriate response to environmental factors [2], an abnormal mucosal immune response in predisposed individuals [14], and the composition of the gut microbiome [5,12,16]. A diet rich in vegetables, fruit, omega-3 fatty acids, and low in omega-6 fatty acids [17], with an adequate intake of dietary fiber, has a protective effect in IBDs [15,17]. It appears that a sufficient serum concentration of vitamin D, which is known for its extensive health-promoting effects [18,19], may also play a protective role [12,20]. The ‘Western diet’ is characterized by high fat consumption, increased intake of proteins and monosaccharides, and highly processed foods, and is linked to a higher risk of developing IBD. This effect is amplified by emulsifiers like carboxymethylcellulose, mentioned in the 2023 ESPEN recommendations, and other additives, including carrageenan, also included in this dietary pattern [14,15,17,21,22]. Due to the increasing consumption of food additives and the observed increase in the incidence of IBD [4,5], our study focuses on investigating the role of food additives with a particular reference to carrageenan. While the number of studies on food additives and IBD is growing, as shown in Figure 1, few focus specifically on the role of carrageenan. This review addresses this gap by critically analyzing recent studies on the effects of carrageenan on gut microbiota, intestinal permeability, and inflammatory processes. By integrating findings from different perspectives, the review provides a holistic view of the need for future research leading to better knowledge with practical benefits for IBD patients.

## 2. Food Additives

**Figure 2:**
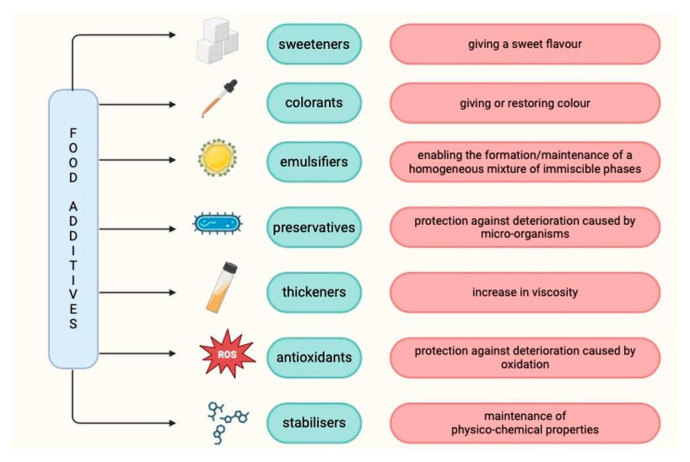
The role of selected food additives based on [23].

Food additives are defined as substances that are not a typical component of food and [23] are added to food to enhance technological capabilities [22,23,24] or to improve flavor or presentation [25]. These include colorings, sweeteners and thickeners, antimicrobials, and antioxidants. Food additives may be added if their use is technologically necessary, safe, not misleading, and beneficial to the consumer [23]. In the European Union, food additives have their own E-number, which is indicated on the product label [26]. The role of selected food additives is shown in Figure 2. 

### 2.1. Food Additives and Autoimmune Diseases

Diet affects the immune system and inflammation, particularly the Western diet with its highly processed foods [27]. The consumption of food additives appears to be associated with an increased incidence of autoimmune diseases [28,29] and inflammation in general. For example, chronic ingestion of polysorbate 80 leads to increased expression of TNF-*α* factor in muscle [30]. However, there are also reports of the anti-inflammatory effects of food additives. For example, sodium benzoate inhibits LPS-stimulated TNF-α and IL-1β expression in mouse microglia [31]. It was also shown that the administration of 80 mg of curcumin daily for 3 months to patients with osteoarthritis led to a decrease in CRP levels (compared to an increase in the placebo group). Moreover, it resulted in a reduction in CD4+ and CD8+ T-lymphocytes and Th17 cells, an increase in the proportion of Treg cells, an increase in FOXP3, and a decrease in the proportion of B-lymphocytes [32]. The effect also appears to cause a loosening of tight junctions and an increase in intestinal permeability (as described later in this article). This may result in the migration of toxins and antigens into the gut, leading to activation of the immune cascade [27,29], as well as increasing the risk of impaired nutrient absorption [27]. 

The evidence highlights the complex role of diet and food additives in modulating immune responses and inflammation. While some additives like polysorbate 80 exacerbate inflammatory processes, others like sodium benzoate and curcumin show anti-inflammatory potential. These findings suggest the dual nature of food additives, warranting further investigation on the mechanisms behind these effects and identifying which additives pose risks versus those that offer therapeutic benefits. 

This appears particularly important considering the diet modifications in patients with autoimmune diseases. 

### 2.2. Food Additives and Other Diseases

Food additives may cause allergic reactions and/or hypersensitivity [33,34,35]. Attention is also drawn to the potentially harmful effects of food additives on children’s health, particularly artificial colors (the ‘Southampton Six’ colors) and/or a sodium benzoate preservative, which may increase hyperactivity in children [36]. Additionally, artificial food colorings appear to exacerbate the symptoms of attention deficit hyperactivity disorder in young adults [37]. 

In mice, it was observed that the intake of emulsifiers (polysorbate 80, soy lecithin, gum arabic, carboxymethylcellulose) at a concentration of 1% led to a reduction in both the weight and length of the colon. However, the results were statistically only significant for polysorbate 80 (reduction in weight and length) and carboxymethylcellulose (length only). No changes were observed for cecal weight. In addition, the intake of emulsifiers resulted in an increase in weight (carboxymethylcellulose and polysorbate 80) and an elevated fasting glucose level (all emulsifiers mentioned above). The authors suggested that carboxymethylcellulose and polysorbate had the strongest effects on inflammatory and metabolic parameters, while soy lecithin and gum arabic had a lesser effect. One limitation of the study was that only the length of the colon was measured, which is considered to be an indirect marker of inflammation [38].

Sweeteners are often associated with impaired carbohydrate metabolism. The results come from both human and animal studies. In humans, saccharin and sucralose increase the glycemic response in a glucose tolerance test (OGTT) compared to glucose; this effect has not been reported for aspartame and stevia [39]. In 10-week-old C57Bl/6 mice, the intake of saccharin, sucralose, and aspartame in drinking water resulted in increased blood glucose levels, with the highest level observed for saccharin. In addition, saccharin caused higher blood glucose concentrations compared to glucose in mice fed a high-fat diet as well as in mice fed a normal diet [40]. Ingestion of 48 mg of sucralose by obese individuals, who did not consume sweeteners by default, resulted in statistically significantly higher glucose concentrations at 60 and 90 min, insulin concentrations at 60, 90, and 120 min, and C-peptide concentrations at 90, 120, and 150 min compared to ingestion of water [41]. Chronic ingestion of polysorbate 80 in mice resulted in elevated plasma insulin levels and increased insulin resistance [30]. In addition, it appears that sodium benzoate may have beneficial effects in the treatment of neurodegenerative diseases [31].

### 2.3. Food Additives and Microbiome

It appears that food additives commonly found in the “Western diet” may be associated with the pro-inflammatory potential of the microbiota [42]. Synthetic sweeteners (including sucralose, saccharin, aspartame, or acesulfame K), other emulsifiers including carboxymethylcellulose, polysorbate 80, and preservatives may have an impact on the gut microbiota [43,44]. In a randomized placebo-controlled study, Chassaing et al. showed that an intake of 15 g/day of carboxymethylcellulose reduced bacterial diversity in the gut (decrease in *Faecalibacterium prausnitzii* and *Ruminococcus* sp., increase in *Roseburia* sp. and *Lachnospiraceae*) and short-chain fatty acid production, and that highly processed foods may cause changes in the gut microbiota, that may lead to inflammation and the development or exacerbation of IBD [45]. In addition, carboxymethylcellulose and polysorbate 80 affect the bacterial taxa *Proteus* spp. and *Veillonellaceae* which have been associated with promoting the recurrence of CD. Moreover, polysorbate 80 and sodium sulphite have an inhibitory effect on the growth of *Faecalibacterium prausnitzii*, which appears to have a favorable effect on CD [46]. Glycerol monolaurate caused intestinal dysbiosis in mice, reduced the content of anti-inflammatory bacteria such as *Akkermansia* and increased levels of LPS [47]. The evidence on the pro-inflammatory potential of the gut microbiome should be interpreted with caution, since most of the studies were conducted on animals. However, reducing bacterial diversity and increasing pro-inflammatory taxa, as seen with carboxymethylcellulose and polysorbate 80, can exacerbate conditions like IBD and CD. Carboxymethylcellulose was listed in the ESPEN guidelines as potentially increasing the risk of IBD [17]. Future research should focus on long-term human studies to better understand the specific impacts of various food additives on gut health [17].

### 2.4. Food Additives and Intestinal Permeability

Increased intestinal permeability leads to bacterial translocation and lipopolysaccharide metabolites into the bloodstream, leading to metabolic endotoxemia and systemic inflammation [48], which is referred to as “leaky gut” [49]. Disruption of the intestinal barrier is seen in many diseases including autoimmune diseases, such as CD, irritable bowel syndrome (IBS), celiac disease, and diabetes [28,29]. Various additives can influence the permeability of the intestine. Decreased surface hydrophobicity of the proximal colon, middle colon, distal colon, and rectum was observed after oral administration of the nonionic detergent Brij 35 to rats and increased epithelial permeability to hydrophilic molecules and increased susceptibility of the mucosa to inflammation after administration of dextran sodium sulphate (DSS) [50]. It has been shown that chronic administration of P80 to mice resulted in an increased LPS in plasma, suggesting increased intestinal permeability [30].

A high-sugar diet in Drosophila melanogaster contributed to increased intestinal permeability, as demonstrated by the so-called “smurf assay”, in which the insect’s blue pigmentation suggests increased intestinal permeability. In addition, the high-sugar diet decreased the length and diameter of the intestine, the overall size of the animals with increased food intake, and decreased the activity of intestinal alkaline phosphatase, which is a protective factor against lipopolysaccharide, reduces inflammation and strengthens tight junctions both in vivo and in vitro [28].

In another study, the intake of emulsifiers and highly processed food among 657 people based on six 24 h dietary recalls was investigated. Antibodies against flagellin and lipopolysaccharide, as well as GlycA levels, were measured. Levels of those parameters were not associated with a higher intake of emulsifiers. In contrast, increased consumption of highly processed foods (%g/d) was associated with higher GlycA levels, and intake of highly processed foods, expressed as %kcal/d, with higher levels of LPS and antibody levels (sum of IgG and IgA for flagellin and LPS). In this case, the association with inflammation and intestinal permeability is due to the highly processed foods, not the emulsifiers themselves [51]. A study with human intestinal epithelial Caco-2 cells showed that sucrose monoester fatty acid loosens tight junctions and increases intestinal permeability, proven by changes in actin filament structure. This surfactant may also increase the paracellular uptake of food allergens [52]. “Leaky gut” plays a crucial role in the pathogenesis of many acute and chronic diseases, often exacerbating their severity. Mechanisms that damage the intestinal barrier can increase patient vulnerability and susceptibility to illness. While these findings are based on hypotheses and experimental studies, they highlight the need for personalized and in-depth dietary advice. Detailed knowledge on food additives included in personalized nutrition may consider individual susceptibility to intestinal permeability and could provide significant clinical benefits.

## 3. Carrageenan

### 3.1. Definition

One food additive is carrageenan, a linear, water-soluble polygalactan that belongs to the group of hydrocolloids [53,54]. It is obtained from the cell walls of red seaweeds, mainly from *Chrondrus crispus*, *Kappaphycus alvarezii*, and *Eucheuma denticulatum* [55]. It is an odorless powder with a white to yellow color [56]. This compound can be obtained in two ways. The first (traditional) method involves extracting the carrageenan from the seaweed, during which it is dissolved and removed. In the second method, carrageenan remains in the seaweed matrix and is not dissolved [55]—resulting in partially refined carrageenan [57]. Carrageenan is labelled with the identification number E407 (refined carrageenan) or E407a (partially refined carrageenan, otherwise processed Eucheuma seaweed) [58]. Processed Eucheuma seaweed contains more cellulose and fewer inorganic salts [59].

Carrageenan comes in six different forms, the most common being: Kappa (κ), Iotta (ι), and Lambda (λ) [60]. They differ in their ability to gel and their solubility in water [61]. The κ form forms strong, stiff gels with potassium salts [57,62] and brittle gels with calcium salts [57], the ι form forms flexible gels with calcium salts, while carrageenan λ has no gelling ability [62] but can be used as a thickening agent [57,62]. In addition, the gel of ι carrageenan is transparent, in contrast to κ carrageenan [57]. All forms of carrageenan are soluble in warm water, but only λ is soluble in cold water [53,61]—this depends on the number of sulphate groups per galactose unit [53].

### 3.2. Carrageenan Market

The intake of carrageenan in a typical Western diet is estimated at 250 mg/person/day but can reach 2–4 g/person/day [63]. This compound is also the fourth most commonly consumed food additive in pediatric patients with CD [64]. The compound annual growth rate (CAGR) for the carrageenan market is estimated to reach 6.5% by 2028, primarily due to the increasing consumption of highly processed foods, but also due to the increasing demand for hydrocolloids. Europe is the fastest-growing market, and the Asia–Pacific region has the largest global market share, due to its significant production and consumption, and its favorable climatic conditions for cultivation and production. South America and Africa are characterized by their small market size [65]. It is estimated that the global market will reach USD 1.32 billion by 2030 [66].

### 3.3. Occurrence

As a food additive, carrageenan has a thickening, emulsifying, stabilizing, and gelling function [60]. It is used in dairy products where, when added in low concentrations, it prevents the separation of whey (κ form) and prevents the separation of protein and fat from milk and fat from cream. The addition of carrageenan (κ or λ) to cream improves the whipping process [57]. One study found that carrageenan is present in 66% of frozen desserts [64] and the largest amount is found in frosting mixes [61]. Another group involves meat products, where carrageenan binds free water and interacts with proteins to create a higher-quality product. The next group are carrageenan-based jellies, which are an alternative to gelatine-based products [57]. In addition to food, carrageenan is also used in cosmetics, animal feed, and in the pharmaceutical industry where it is used as a foaming, dissolving, or stabilizing agent [56,67]. It is used in the formulation of pharmaceutical capsules instead of gelatine and in biosensors. Carrageenan’s properties and advantages over synthetic polymers (including high availability and biodegradability) have also led to its use as an industrial material in the form of nanocomposites which eliminate pollution from the environment, functional textiles, as well as for controlled release technologies for fertilizers [67]. It can also be used in tissue engineering as a carrier matrix to transfer cells [68].

### 3.4. Safety of Carrageenan as a Food Additive

Carrageenan is widely regarded as safe. Its use as a food additive has been approved by the European Food Safety Authority (EFSA), the Joint FAO/WHO Expert Committee on Food Additives (JECFA) as well as the Food and Drug Administration (FDA) [58,69,70] (Table 1). However, the National Organic Standards Board (NOSB) in 2016 withdrew carrageenan from the list of substances permitted for use in organic foods. The report listed environmental contamination during the production of carrageenan, mentioning the fact that the function of carrageenan can be achieved using other additives such as xanthan gum, gellan gum, or guar gum. Another reason is its lack of compatibility with the definition of sustainable agriculture [71]. 

### 3.5. Impact on Inflammatory Bowel Diseases

Degraded carrageenan was used in the last century to induce intestinal ulceration in animals [72,73,74]. Their severity increased with the duration of supply of this compound [72]. The administration of a 5% solution of degraded carrageenan to guinea pigs in drinking water resulted in decreased body weight (loss of 15–25% compared to the beginning of the study), the appearance of blood in the feces (occult in all animals after 30 days and visible in one animal after 45 days of the study) and loose stools already at the end of the first week of the study [72]. Another study showed that administration of a 10% degraded carrageenan solution to mice resulted in bloody diarrhea and perianal inflammation, while weight changes were not significant. Histopathologically, deformations of the crypts and cellular inflammatory infiltrations in the lamina propria were observed [73]. Other studies have also been conducted with other animals including rabbits [74] and rats [75].

It was already noted in the previous century that the changes induced by degraded carrageenan in animals are in some ways similar to those found in UC in humans [76]. These reports were also confirmed in later years [77]. In 2016 Muynaka et al. described the changes induced by administrating of a 1% medium molecular weight carrageenan solution in drinking water to piglets. Swelling of the mucosa and submucosal layers of the intestines was observed, but without granulomatous inflammation, similar to the symptoms associated with UC, as the authors hypothesized [77].

Only a few human studies are looking at the effect of carrageenan on IBD. One randomized placebo-controlled study from 2017 investigated the effect of a carrageenan-free diet on UC activity. At the beginning, participants were instructed to follow a carrageenan-free diet. They were then randomized to take 100 mg carrageenan capsules (study group) or dextrose capsules (placebo group). The main outcome was the occurrence of relapse, i.e., an increase in at least two points on the Simple Clinical Colitis Activity Index (SCCAI) scale, along with an increase in the dose of maintenance medication or the introduction of a new medication for the patient if UC symptoms worsened. After the intervention, SCCAI scale scores increased by at least two points in three patients taking carrageenan capsules and in one patient in the placebo group (without increasing the dose of medication). Relapse was observed in three patients in the study group, two of whom were already taking medication. The study was completed by four patients in the placebo group and two patients in the carrageenan group without relapse. There was also a decrease in the SCCAI scale in four subjects in the group taking the dextrose capsules. In addition, an increase in IL-6 and fecal calprotectin levels was observed in subjects taking carrageenan capsules, while TNF-α levels decreased in two subjects in the control group and one subject in the study group. The results suggest that carrageenan may contribute to the development of UC relapses. It should also be pointed out that the doses of carrageenan used in this study were lower than those consumed in the “Western diet” [63].

In 2018, a study was published that investigated exposure to food additives among children and young adults with CD. The 24 h dietary recall of patients was used, from which a database of all food products was created and the intake of eight food additives, including carrageenan, was checked. The consumption of food additives was found to be widespread in this group, particularly xanthan gum, maltodextrin, soy lecithin, and carrageenan [64]. Moreover, after screening 116,087 adult participants aged 35–70 years, it was shown that a higher intake of ultra-processed foods (soft drinks, salty snacks, processed meat, refined sweetened foods) positively correlated with a higher risk of an IBD incident [78]. It has also been shown that a diet low in emulsifiers can have a positive impact on the symptoms associated with CD and is perceived as appetizing by most respondents [38].

In 2020, Sandall et al. published a study in which they investigated the feasibility and acceptability of a low-emulsifier diet, meaning that it excludes 65 food additives considered to be emulsifiers, in 20 patients with CD in remission. The subjects adhered to this diet for 14 days and evaluation was based on 7-day food diaries kept 7 days before the start of the study and during the last 7 days of the intervention. Prior to this, subjects received informational brochures containing products suitable and unsuitable for their diet, dietary counseling, and access to a dedicated app to help identify products containing emulsifiers. In this study, emulsifier intake was assessed by the frequency of consumption of products containing emulsifiers. Before the beginning of the study, 15/20 participants consumed emulsifiers daily, while the remaining 5 participants consumed emulsifiers on 6 of the 7 days in the week under evaluation. In total, 19 of 20 subjects followed the low-emulsifier diet well (reducing the frequency of emulsifier consumption by at least 75% between baseline and the end of the intervention). A total of 18 subjects declared following the diet for the entire study period, and two subjects for ¾ of the time, due to the need to have food away from home. Although 90% of the subjects rated the low-emulsifier diet as more difficult to follow than their usual diet, 95% found it appetizing. In addition, there was an improvement in symptoms of CD assessed by PRO-2 [38].

Carrageenan may have pro-inflammatory effects. Oral administration of processed Eucheuma seaweed to rats led to deformation and destruction of intestinal villi, the presence of inflammatory infiltration of the small intestinal lamina propria and a decrease in the amount of goblet cells. In addition, eosinophils, macrophages, and lymphocytes were present in the intestinal lamina propria. This was consistent with an increase in inflammatory parameters, i.e., CRP and middle molecules [79]. It should be emphasized that in this study processed Eucheuma seaweed were used, which has been considered as safe and approved for use in food [58]. Carrageenan can also cause an increase in the activity of the transcription factor NF-κB in the intestinal epithelium, which results in an increased expression of the proinflammatory IL-8 gene [80] but another study showed that this only occurs in the presence of LPS [81]. In addition to this, there is an unbeneficial change in the distribution of tight junctions and a decrease in the overall density of ZO-1 protein [80]. It should be noted that most conclusions come from studies on cell lines. ZO-1 can be used as a marker of tight junction integrality in future studies on the effects of food additives on intestinal permeability.

Table 2 summarizes the results of animal studies. A summary of human studies focusing on the effects of carrageenan on IBD and the impact of the food additives is presented in Table 3. 

### 3.6. Impact on the Microbiota

The microbiota of IBD patients differs from that of healthy individuals and is characterized by a decrease in *Firmicutes* [46], *Bacteroidetes*, *Lactobacillus*, and *Eubacterium* [14], and an increase in *Proteobacteria* [46,82] and *Enterobacteriaceae* including *Escherichia coli* and *Fusobacterium* [13,14]. The intake of carrageenan can lead to changes in the gut microbiota and reduce bacterial diversity. Mi et al. demonstrated that administration of κ carrageenan in drinking water to C57BL/6J pathogen-free mice can exacerbate colitis while on a high-fat diet. A decrease in the anti-inflammatory *Akkermansia muciniphila* bacteria and an increase in pathogenic bacteria have been suggested as possible causes [83]. On the other hand, Wu et al. showed that the supply of κ carrageenan increased the total bacterial abundance and did not decrease microbiota diversity [84]. 

A reduction in *Akkermansia muciniphila* after administration of carrageenan was also observed in other studies [84,85]. *Akkermansia muciniphila* has been negatively correlated with serum TNF-α levels and plays an important role in maintaining intestinal homeostasis. It appears to have a protective effect on inflammatory diseases progressing with disruption of the intestinal barrier, including carrageenan-induced colitis [85].

Moreover, the intake of different types of carrageenan may affect other bacterial species. For example, ι carrageenan contributes to a reduction in *Faecalibacterium* [86] and κ carrageenan caused a decrease in *Proteobacteria* and *Firmicutes* [84,85]. It has been shown that low levels of *Faecalibacterium* spp. may be associated with higher risk of CD recurrence [46]. 

Given the variable effects of carrageenan on the intestinal microbiota observed across different studies, further research is essential to derive conclusive insights on this topic.

### 3.7. Carrageenan and Cancer

Cancers are one of the leading causes of premature death worldwide. They are estimated to be a cause of premature death in 57 countries in 2019, mainly in Canada, the United States, China, Australia, Peru, Argentina, Chile, Colombia, and Western Europe [87]. Globally, breast cancer is the most common, followed by prostate cancer, then lung cancer and colorectal cancer [88]. There are many types of anti-cancer therapies, including chemotherapy, radiotherapy, immunotherapy, and surgical treatment [89]. Many of these therapies result not only in the destruction of cancer cells, but also of healthy cells, which adversely affects the patient’s general condition and limits the effectiveness of the treatment [89,90]. Some studies have shown that carrageenan can inhibit cancer cell growth by arresting the cell cycle in specific phases and delaying cell cycle progression [91]. Others have shown decreased cell viability in tumor zones [92,93] and increased immune responses [94]. The cytotoxic effects of carrageenan may correlate with the number of sulphate groups which may be a result of its higher antioxidant activity [91,93] but the data are inconsistent because it has also been shown that the presence of these groups can inhibit cytotoxic effect [95]. The impact of carrageenan in the context of cancer is presented in Table 4.

Noteworthily, there are also reports that carrageenan has no cytotoxic effects on healthy cells, but only on cancer cells [91].

On the other hand, administration of 15% undegraded carrageenan (Viscarin 402) to female F344 rats was shown to increase the incidence of colon tumors induced by subcutaneously injected azoxymethane or rectally administered methylnitrosourea [99]. Additionally degraded κ carrageenan increased the incidence of high-grade dysplasia and tumor incidence in a mouse model of azoxymethane-induced colorectal cancer [100]. Future studies should focus on well-designed animal models and human trials to evaluate the safety and efficacy of carrageenan in cancer therapy. It will be critical to understand the exact mechanisms by which carrageenan interacts with cancer cells and its long-term effects on healthy and cancerous tissue. This research will help determine whether carrageenan can be safely integrated into cancer therapy or whether its use should be restricted to avoid potential adverse effects.

### 3.8. Impact on Other Diseases

In addition to its effects on inflammation, cancer and IBD, carrageenan has also been shown to have effects on other diseases. The administration of carrageenan to mice in drinking water can lead to increased glucose intolerance compared to a control group of mice that did not receive this additive, additionally leading to higher insulin levels and greater insulin resistance. The authors suggested that this compound may increase the risk of diabetes [101]. There have also been reported cases of IgE-mediated allergy after carrageenan supply [102,103]. There are also reports that ι carrageenan can inhibit the spread of murine cytomegalovirus [104], carrageenans λ, κ, and ι may have a protective effect against herpes simplex virus type 2 (HSV-2) infection in mice [105]. Carrageenan-based gels (Carraguard) have also been tested for efficacy, safety, and acceptability in the context of human immunodeficiency virus (HIV). It appears to be safe and acceptable, but there is evidence that it is not effective in preventing HIV infection [106,107,108,109]. It was shown that κ carrageenan extracted from *Hypnea musciformis* exhibited antibacterial activity against *Staphylococcus aureus* and antifungal activity against *Candida albicans*. Furthermore, in vitro, the same compound showed neuroprotective effects in 6-hydroxydopamine-induced neurotoxicity in human neuroblastoma cells [97].

## 4. Carrageenan, Degraded Carrageenan, and Poligeenan—Differences

It is important to note the difference between carrageenan, degraded carrageenan, and poligeenan. Poligeenan is formed from carrageenan during acid hydrolysis at pH 0.9–1.3 and temperature > 80 degrees Celsius under laboratory conditions. The term ‘degraded carrageenan’ currently refers to the products of the test material used in nutritional research. Both compounds are formed under laboratory conditions and are not part of the molecular weight profile of carrageenan. All of the above compounds have different molecular weights. Poligeenan has a low molecular weight, i.e., 10,000–20,000 Da, degraded carrageenan an average of 20,000–40,000 Da, while the carrageenan used in food products has a high molecular weight (200,000–800,000 Da). As suggested by the authors of the above review, confusing nomenclature causes problems in the precise understanding of studies [62] which was also noted during the writing of this paper. It is necessary to pay particular attention to the nomenclature and to distinguish between carrageenan found in food products and degraded carrageenan and poligeenan.

## 5. Conclusions

**Figure 3:**
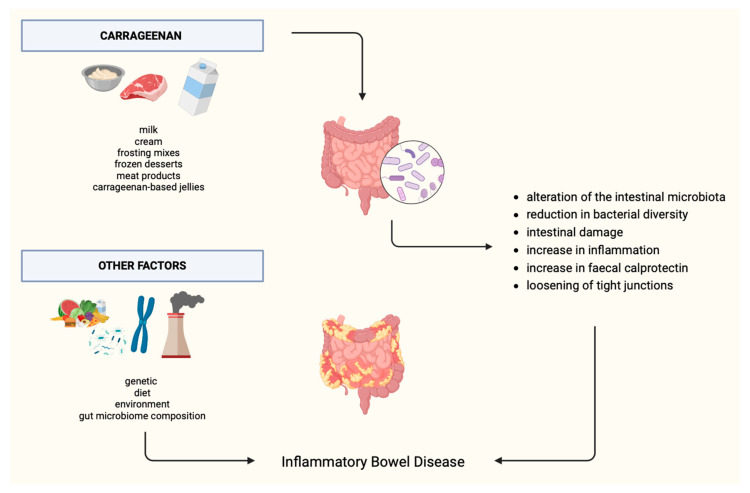
Effect of carrageenan on IBD.

Carrageenan is a commonly used food additive that has been generally regarded as safe. However, recently there have been new studies assessing the effects of carrageenan on health, including IBD. Potentially pro-inflammatory properties, increasing intestinal permeability, impairing glucose tolerance, and the composition of the intestinal microbiome were observed. The effect of carrageenan on IBD is presented in Figure 3. On the other hand, there are studies suggesting that carrageenan may inhibit the growth of cancer cells; however, more studies are needed to determine the role of this additive in oncogenesis. Based on the available literature, it can be concluded that additional, particularly randomized clinical trials are needed to answer whether carrageenan (and other food additives) should be excluded from the diet of individuals exposed to and/or suffering from IBD. In addition, special attention should be paid to the exact nomenclature of carrageenan when writing scientific papers or constructing studies. Making a distinction between carrageenan presented in food, degraded carrageenan, and polygeenan is essential. The research should focus on examining both the effects of degraded carrageenan and carrageenan that is approved for human consumption.

## Figures and Tables

**Table 1:** Reviews provided by selected organizations on the safety of carrageenan.

Organisations	Conclusions	References
EFSA	No adverse effects were found in the context of carcinogenicity or genotoxicity. An ADI = 75 mg/kg b.w. was found to be temporary. However, degraded carrageenan cannot be used as a food additive.	[58]
JECFA	“The use of carrageenan in infant formula or formula for special medical purposes at concentrations up to 1000 mg/L is not of concern”.	[69]
FDA	Carrageenan can be used as a food additive and is considered safe under certain conditions, including that it is a hydrocolloid formed from red seaweed by extraction and that it is used as a stabilizing, emulsifying, thickening substance in the necessary amount.	[70]

**Table 2:** Animal studies reporting effects of carrageenan on IBD.

Carrageenan Solution	Type of Animals	Result	References
1% medium molecular weight carrageenan solution	Piglets	swelling of the mucosa and submucosal layers without granulomatous inflammation	Munyaka et al. Front Microbiol, 2016 [77]
5% solution of degraded carrageenan	Guinea pigs	decreased body weight; appearance of blood in the feces and loose stools; ulcers in large intestine	Watt et al. Gut, 1971 [72]
10% degraded carrageenan solution	Mice	bloody diarrhea; perianal inflammation	Fath et al. Digestion, 1984 [73]
1% degraded carrageenan solution	Rabbits	visible fecal blood; cecal ulceration	Al-Suhail et al., Histochem J, 1984 [74]
5% solution of degraded carrageenan	Rats	cecal ulceration	Marcus et al. Lancet, 1971 [75]
processed Eucheuma seaweed	Rats	destruction of intestinal villi, the presence of inflammatory infiltration of the small intestinal lamina propria and a decrease in the amount of goblet cells	Pogozhykh et al. Int J Mol Sci, 2021 [79]

**Table 3:** A summary of human studies focusing on the effects of carrageenan on IBD and the impact of food additives.

Study Group	Outcome/Conclusion	References
12 adults with UC	Carrageenan supply may contribute to earlier relapse in patients with ulcerative colitis in remission	Bhattacharyya et al. Nutr Healthy Aging, 2017 [63]
138 children with CD	Children with CD frequently consume food additives, particularly xanthan gum, maltodextrin, soy lecithin, and carrageenan	Lee et al. Dig Dis Sci, 2018 [64]
116,087 adults	Higher intake of ultra-processed foods positively correlated with a higher risk of an IBD incident	Narula et al. BMJ, 2021 [78]
20 patients with CD in remission	90% of subjects rated the low-emulsifier diet as more difficult to follow than their usual diet, 95% found it appetizing; low-emulsifier diet led to improvement of symptoms	Sandall et al. Nutrients, 2020 [38]

**Table 4:** Summary of the impact of carrageenan on cancer.

Type of Carrageenan	Cell Type	Mechanism and/or Effect	Study
κ and λ	cervical carcinoma cell lines HeLa	stopping the cell cycle in G1 (only λ carrageenan) and G2 phases (λ and κ carrageenan), delaying cycle progression and consequently inhibiting tumor cell growth	Prasedya et al. BMC Complement Altern Med, 2016 [91]
extracted from *Gigartina pistillata*	colorectal cancer stem cells	reduction in cell viability in tumor zones	Cotas et al. Mar Drugs, 2020 [93]
λ	breast cancer cells 4T1 and melanoma cells B16-F10	reduction in tumor weight and volume and increasing the tumor immune response by raising more activated CD4+ and CD8+ T-lymphocytes in the spleen and M1 macrophages that infiltrate the tumor (after intratumoral injection)	Luo et al. Sci Rep, 2015 [94]
κ	malignant breast cancer cell lines MCF-7	decrease in cell viability, potential apoptotic effect	Sayın et al. Aegean J Med Sci, 2022 [92]
λ extracted from *Laurencia papillosa*	breast cancer cell line MDA-MB-231	decrease in cell viability, inhibition of cell proliferation, regulating genes involved in apoptosis, cell growth in the sub-G1 phase	Jazzara et al. Int J Cancer Manag, 2016 [96]
κ extracted from *Hypnea musciformis*	breast cancer cell line MCF-7 and neuroblastoma cell line SH-SY5Y	reduction in proliferative capacity, but lack of cytotoxic effects	Souza et al. Int J Biol Macromol, 2018 [97]
κ- and λ-from *Chondrus armatus* and low-molecular weight degradation products	esophageal cancer cell lines KYSE30 and FLO1	cytostatic activity (higher for low-molecular weight degradation products), monocytes induction to produce pro-inflammatory cytokines, induction of anti-inflammatory cytokine secretion (observed only for a λ carrageenan low-molecular weight degradation product)	Cicinskas et al. J Biomed Mater Res A, 2020 [98]

## Abbreviations

ADI	adequate daily intake
CAGR	compound annual growth rate
CD	Crohn’s disease
CRP	C-reactive protein
DSS	dextran sodium sulphate
EFSA	European Food Safety Authority
ESPEN	European Society for Clinical Nutrition and Metabolism
ESR	erythrocyte sedimentation rate
FDA	Food and Drug Administration
HIV	human immunodeficiency virus
HSV-2	herpes simplex virus type 2
IBD	inflammatory bowel disease
IBS	irritable bowel syndrome
JECFA	Joint FAO/WHO Expert Committee on Food Additives
LPS	lipopolysaccharide
NF-κB	nuclear factor-κB
NOSB	National Organic Standards
OGTT	oral glucose tolerance test
SCCAI	Simple Clinical Colitis Activity Index
TNF-α	tumor necrosis factor α
UC	ulcerative colitis
ZO-1	zonula occludens-1
